# Notes and News

**Published:** 1894-09-08

**Authors:** 


### Notes and News.

Collected and Communicated.

The cholera shows no signs of spreading rapidly anywhere, although new cases appear in small numbers in fresh districts. The latest infected locality is Flushing where four sailors have been attacked by the disease. In the meanwhile, quarantine stations are multiplying, and those already on the Continent are doubtlessly concerned as to their chances of an unpleasant experience, the dread of which will also deter some would-be travellers from leaving home at present.

Again we have to record another instance of Mr. Passmore Edwards' generosity and interest in the care of the sick. Last week this gentleman laid the foundation-stone of a cottage hospital at Wood Green, the cost of which he is defraying himself, and which will amount to about £2,000. The hospital is to contain two wards, male and female, containing each four beds. There is to be an operating-room and convenient quarters for the nursing staff, and the usual offices. The event of laying the foundation-stone created much interest locally, and entertainments for the benefit of the hospital were organised by the trades unions of the district.

An unusually large number of governors attended the quarterly general court of the Middlesex Hospital held on August 30th, under the presidency of Mr. J. W. Hulke, F.R.S., President of the Royal College of Surgeons. The report showed a continued increase of subscriptions, whilst the donation list was chiefly noteworthy for the princely gift of £1,000 by Mr. John Maple, of Bedford Lodge, Hampstead. The list of legacies contained several items of importance, amongst which may be mentioned the bequest of £434 by Mr. T. H. Gleeson, in respect of which Mr. Henry T. Gleeson was appointed an honorary governor. The expenditure of £18,475 was sanctioned for the construction of the convalescent home at Clacton-on-Sea, the building of which has already commenced. The home is to contain forty beds for the use of the patients of the hospital. Subsequently the court proceeded to make a slight alteration in the laws of the hospital, in order to allow of the appointment of a third treasurer, and the Hon. Lionel Walter de Rothschild was elected to the newly-created office.

In this age in which social and medical questions are becoming more and more connected such a Congress as that which is to be held in Paris in June next cannot fail to arouse general interest. This Congress is the Penitentiary Congress, held for the purpose of the comparison and study of criminology. The Congress is to consist of delegates of various Governments, prison officials of high rank, delegates from societies, experts in criminology, various distinguished persons and others interested in the subject of the Congress. The Congress will be divided into four principal sections: (1) Penal Legislation; (2) Penitentiary Institutions; (3) Preventive Institutions; and (4) Questions Relative to Children and Minors. It is stated that England is to take no part in the Congress, a resolution that has caused some surprise in Paris. June is yet far off, and a different resolution may yet be arrived at.

The leper community of Robbin Island have always enlisted public interest, and to some extent this has been effected by personal interest in individuals who have given up their lives to work amongst them. A clergyman writes now to the papers that Robbin Island no longer elicits tangible spmpathy, and that with the death of Father Damien funds have ceased to flow towards it. But it has been pointed out, and not for the first time, that lepers in India are in a far worse plight, and that, moreover, they are a danger to the whole population, native or otherwise. The separation of lepers from the healthy population appears to be the only known effectual means of stamping out the disease. Strenuous effort, and agitation even, may fairly be directed towards the proper care of at least 126,000 afflicted, who are returned as lepers, in India, Surely this is a greater curse to a country than any opium trade, and now that would-be reformers have had the satisfaction of securing a hearing of facts before a commission, they might find something to do by turning their minds to solving the problem of how to stamp out leprosy in India.

Saturday will be a day of great interest in Birmingham, when the foundation-stone of the new General Hospital is to be laid by the Duke of York. It will be remembered that the scheme received its first impetus from the fact that a munificent bequest of £25,000 was received from Miss Ryland, which required that new accommodation for the in-patients of the old General Hospital should be provided within five years of her death. The sum required to secure an institution adequate in every way for so important a centre as Birmingham has proved far greater than was expected. The site alone has absorbed £48,000; the architect's commission £7,500; the actual buildings are estimated at £117,888, the foundations at £5,000. Then there is the furnishing, machinery, and other necessary expenses to account for, making an estimated total of £206,000. The various amounts already secured leave £50,000 yet to be provided. It may be interesting to add that, exclusive of land and fittings, the Royal Infirmary, Liverpool, cost £386 per bed, whereas the new General Hospital is estimated to cost £360 per bed. The Royal Infirmary, Liverpool, has 270 beds, and Birmingham General Hospital 341. There is no Nurses' Home at Liverpool as at Birmingham.

